# Two new virtual reality tasks for the assessment of spatial orientation Preliminary results of tolerability, sense of presence and usability

**DOI:** 10.1590/1980-57642018dn12-020013

**Published:** 2018

**Authors:** Raquel Quimas Molina da Costa, José Eduardo Pompeu, Daniel Donadio de Mello, Emerson Moretto, Fernanda Zillig Rodrigues, Michelle Didone dos Santos, Ricardo Nitrini, Francesca Morganti, Sonia Maria Dozzi Brucki

**Affiliations:** 1 Departamento de Neurologia, Faculdade de Medicina da Universidade de São Paulo, SP, Brazil.; 2 Departamento de Fisioterapia, Fonoaudiologia e Terapia ocupacional da Faculdade de Medicina da Universidade de São Paulo, SP, Brazil.; 3 Departamento de Engenharia de Sistemas Eletrônicos, Escola Politécnica, Universidade de São Paulo, SP, Brazil.; 4 Dipartimento di Scienze umane e sociali, Università degli studi di Bergamo, Bergamo, Italy.

**Keywords:** spatial orientation, ecological momentary assessment, immersive virtual reality, technology assessment, biomedical, user-computer interface, orientação espacial, avaliação momentânea ecológica, realidade virtual imersiva, avaliação de tecnologias em saúde, interface usuário-computador

## Abstract

Spatial orientation is a cognitive domain frequently impaired in Alzheimer's Disease and can be one of its earliest symptoms.

**Objective:**

This paper describes the results of tolerability, sense of presence and usability of two immersive virtual reality tasks for the assessment of spatial orientation, using VR headset in adults.

**Methods:**

31 healthy adults recruited from university and the local community performed two experimental immersive virtual reality tasks of spatial orientation: the SOIVET-Maze for the assessment of allocentric to egocentric spatial abilities and the SOIVET-Route for the assessment of spatial memory and landmark recognition. Participants completed questionnaires about sense of presence, cybersickness symptoms, technology use profile and motion sickness history. Usability measures were assessed by spontaneous feedback from participants.

**Results:**

All participants were able to understand the task instructions and how to interact with the system. Both tasks seemed to induce a strong sense of presence, as assessed by the Witmer and Singer Presence Questionnaires (M=128 and 143 for SOIVET-Maze and SOIVET-Route, respectively). The SOIVET-Route had a small numeric advantage over the SOIVET-Maze tolerability scores assessed by the Cybersickness Questionnaire (M=4.19, SD=5.576 and M=3.52, SD=6.418 for SOIVET-Maze and SOIVET-Route respectively). Also, there were no drop-outs on the SOIVET-Route due to tolerability issues, unlike the SOIVET-Maze, which had two drop-outs. However, this difference was not statistically significant (Z= -.901, p= 0.368, Wilcoxon signed-rank test).

## Resumo

A orientação espacial é um domínio cognitivo freqüentemente comprometido na doença de Alzheimer e pode ser um dos primeiros sintomas manifestados.

**Objetivo:**

Este artigo descreve os resultados de tolerabilidade, sensação de presença e usabilidade de duas tarefas imersivas de realidade virtual para avaliação da orientação espacial, utilizando óculos de RV em adultos.

**Métodos:**

31 adultos saudáveis, ​​recrutados entre estudantes universitários e da comunidade local, realizaram duas tarefas de realidade virtual imersiva para avaliação da orientação espacial: A tarefa SOIVET-Maze para avaliação da capacidade de transposição da orientação alocêntrica para egocêntrica e a tarefa SOIVET-Route para avaliação da memória espacial e reconhecimento de pontos de referência. Os participantes também responderam questionários sobre Sensação de Presença, Sintomas de *cybersickness,* Perfil de Uso de Tecnologia e Histórico de cinetose. Feedback espontâneo dos participantes foi utilizado como medida de usabilidade.

**Resultados:**

Todos os participantes conseguiram compreender as instruções da tarefa e como interagir com o sistema. Ambas tarefas parecem induzir forte sensação de presença, avaliada pelo Questionário de Presença de Witmer e Singer (M=128 e 143 para SOIVET-Maze e SOIVET-Route, respectivamente). A tarefa SOIVET-Route teve uma pequena vantagem numérica em relação à tarefa SOIVET-Maze na pontuação de tolerabilidade avaliada pelo Questionário de *Cybersickness* (M=4,19, SD=5,576 e M=3,52, SD=6,418 para SOIVET-Maze e SOIVET-Route respectivamente). Além disso, não houve desistências na SOIVET-Route devido a problemas de tolerabilidade, ao contrário da SOIVET-Maze, que teve dois *drop-outs.* No entanto, essa diferença não foi estatisticamente significativa (Z= -901, p=0,368, teste de postos sinalizados de Wilcoxon)

Spatial orientation is the ability of finding our way in familiar or unfamiliar surroundings.^1^ It involves two major aspects of spatial cognition: Egocentric orientation, which provides spatial information from the viewpoint of the navigator, and Allocentric orientation, involving focus on the spatial relationship between landmarks, independent of the navigator's position.^1^ Deficits in spatial orientation are a common manifestation of Alzheimer's Disease (AD) and can be one of its earliest symptoms.^1^

Several studies have described spatial orientation deficits in patients with a measurable decline in cognitive abilities, yet without impairment in daily functioning, a condition known as Mild Cognitive Impairment (MCI).^2^^-^ 5 These deficits have been shown to correlate with hippocampal and parietal atrophy in AD and MCI patients, as well as with a greater risk of progression from MCI to AD.^2^^-^ 5 Such findings suggest that a decline in spatial abilities may represent a prodromal stage of an underlying degenerative process, and studies have supported the inclusion of spatial orientation assessment in elderly and MCI patients.^6^^-^ 8

However, there is still no gold standard for the assessment of spatial orientation abilities and several new tasks have been proposed over the years.^2^^,^ 9 ^,^ 10 Traditional paper-and-pencil testing usually lacks ecological validity to assess different spatial orientation components, such as landmark recognition, mental rotation, egocentric and allocentric abilities, that can be diversely impaired in elderly and MCI patients.^5^^,^ 11 ^,^ 12 Moreover, patient performance on small-scale spatial orientation tasks correlates only partially with performance on large-scale spatial tasks.^11^ This may indicate that different cognitive abilities are recruited depending on different scale perceptions.^5^

Recently, virtual reality (VR) environments have provided new possibilities for the ecological assessment and rehabilitation of cognitive deficits in the elderly population.^12^^-^ 14 In particular, VR tasks for the assessment of spatial orientation have shown to be a valid and feasible tool, but most of these tasks were not developed for immersive environments.^11^^,^ 12 ^,^ 15 Among VR environments, immersive interactive tasks appear to have advantages for ecological cognitive assessment and rehabilitation, as they can reproduce real-world sensory perceptions.^14^^,^ 16 Immersive environments are becoming increasingly available and have shown some promising results for different age-related declines, such as sensory, motor and cognitive decline.^17^^-^ 19 However, the focus of most immersive virtual reality tasks used with elderly has been on motor or cognitive rehabilitation rather than assessment.^14^^,^ 16 ^,^ 19

One remaining restriction of immersive virtual reality - whether for therapeutic or entertainment purposes - is cybersickness, a form of motion sickness that promotes a dizzy feeling that occurs while performing or after stopping the task or game; in some cases leading to nausea, vertigo and vomiting.^19^ Cybersickness is one of the reasons why fewer studies have focused on immersive virtual environments for elderly, along with greater difficulty coping with computerized systems, engagement in rigid procedures and age-related physical and cognitive limitations.^19^

Following the current progression of spatial orientation assessment and the advantages of immersive virtual interactive tasks, our group has focused on developing two virtual reality tasks using a new proposed system, the Spatial Orientation in an Immersive Virtual Environment Test (SOIVET): SOIVET-Maze task and SOIVET-Route task. In order to evaluate User Experience, this study aims to describe preliminary results of the SOIVET: tolerability, sense of presence, and usability of both tasks in adults.

## Methods

### Study sample

This pilot study was a cross-sectional observational study conducted at the Reference Center for Cognitive Disturbances of the University of São Paulo Clinicas Hospital, in São Paulo, Brazil and approved by the institution's Ethics Committee (Comitê de Ética do Hospital das Clínicas da Faculdade de Medicina da Universidade de São Paulo - CEP USP, reference number 2.319.633). A total of 31 healthy adults recruited from both university and the local community participated in this study. Eligible participants had to be 18-59 years old, have normal or corrected eyesight and no history of cognitive or hearing impairment. None of the participants had previously performed any of the proposed tasks. Technology use profile (Box 1) and motion sickness history (Box 2) were assessed with specific questionnaires developed by our research group, and were not used for eligibility. Physical comorbidities were scored using the Charlson Comorbidity Index (CCI). Informed consent was given by all participants.

**Box 1:** Questions of the Technology Use Profile Questionnaire.

Questions in Portuguese	Questions in English
1. Com que frequência você utiliza o computador?	1. How often do you use a computer?
2. O seu celular é do tipo smartphone ou IPhone?	2. Is your cell phone a smartphone or an iPhone?
3. O seu celular tem acesso à internet ?	3. Does your cell phone have internet access?
4. Com que frequência você utiliza a internet do celular?	4. How often do you use the internet on your cell phone?
5. Você tem familiaridade com a tecnologia *touchscreen*?	5. Are you familiar with touchscreen technology?
6. Você joga algum jogo no celular ou no tablet?	6. Do you play any games on your cell phone or tablet?
7. Com que frequência você joga no celular ou no tablet?	7. How often do you play on your cell phone or tablet?
8. Você joga ou jogava algum videogame?	8. Do you play or did you use to play any videogames?
9. Com que frequência você joga ou jogava videogame?	9. How often do you play or did you use to play videogames?
10. Você costuma utilizar aplicativos de GPS no celular ou no carro?	10. Do you usually use GPS applications on your cell phone or in your car?

**Box 2:** Questions of the Motion Sickness Screening Questionnaire*.

Questions in Portuguese		Questions in English
1. Você está sentindo algum desconforto neste momento? Se sim, por favor descreva.		1. Are you feeling any discomfort right now ? If yes, please describe.
2. Você teve episódios de vômito ou enjôo hoje ou nos últimos dois dias?		2. Did you have any episode of vomiting or feel nauseous today or in the last two days?
3. Você tem histórico de enjôo relacionado a algum meio de transporte?		3. Do you have a history of motion sickness related to a mode of transportation?
3.1. Se sim, por favor descreva onde (no carro, em barcos, trens, avião).		3.1. If yes, please describe where (in the car, on boats, trains or airplane).
3.2. Sem sim, por favor descreva quando (recentemente, há muito tempo ou na infância).		3.2. If yes, please describe when (recently, long ago, in childhood)
4. Você já sentiu tontura ou náuseas enquanto assistia a um filme em uma tela grande (ex.: cinema comum, cinema 3D)?		4. Have you ever felt dizzy or nauseous while watching a movie on a big screen (e.g. movie theater, 3D cinema)?
5. Você sente enjôo ou tontura quando lê em um carro ou ônibus em movimento?		5. Do you feel nauseous or dizzy while reading in a moving car or bus?
6. Você prefere ser o motorista, ao invés do passageiro, porque senão você sente tonturas ou náuseas?		6. Do you prefer to be the driver rather than the passenger, because otherwise you feel dizzy or nauseous?

* To be administered prior to VR task performance.

### User Experience outcomes

Tolerability results were assessed with a self-report cybersickness questionnaire based on the validated Brazilian Portuguese version of the Simulator Sickness Questionnaire, which included 16 adverse events related to motion and virtual sickness - including nausea, headache, blurred vision, vertigo - on a 4-item Likert scale of intensity (Not at all - a Little - Somewhat and Very much)^20^^,^ 21 (Box 3). Participants completed the questionnaire after each task. In order to investigate the possibility of an individual vulnerability component for cybersickness, participants were given a short screening questionnaire for motion sickness history, prior to task performance. The screening questionnaire was developed by our research group and included 6 questions to screen situations that could induce motion sickness: “Do you feel dizzy or nauseous while reading in a moving car or bus?” or “Do you prefer to be the driver rather than the passenger, because otherwise you feel dizzy or nauseous?” (Box 2).

**Box 3:** Cybersickness Questionnaire*.

Intructions in Portuguese					Instructions in English				
Por favor, marque o quanto você sentiu qualquer um dos sintomas abaixo:					Please indicate how strongly you felt any of the symptoms listed below:				
Sintomas	Nem um pouco	Um pouco	Bastante	Muito	Symptoms	Not at all	A little	Somewhat	Very much
Desconforto geral					General discomfort				
Cansaço					Fatigue				
Dor de cabeça					Headache				
Vista cansada					Eyestrain				
Aumento da salivação					Increased salivation				
Suor					Sweating				
Náusea					Nausea				
Dificuldade de concentração					Difficulty concentrating				
Taquicardia					Tachycardia				
Visão borrada					Blurred vision				
Tontura (com olhos abertos)					Dizziness (with eyes open)				
Tontura (com olhos fechados)					Dizziness (with eyes closed)				
Confusão mental					Mental confusion				
Vertigem					Vertigo				
Desconforto abdominal					Stomach discomfort				
Arroto ou refluxo					Burp or reflux				

* Based on the Brazilian version of the Simulator Sickness Questionnaire^21^.

Sense of presence was investigated using the Witmer and Singer Presence Questionnaire.^22^ This questionnaire was proposed in 1998 by Bob G. Witmer and Michael J. Singer and was based on the hypothesized factors that contribute to one's sense of presence: control, sensory, distraction and realism factors.^22^ Participants were asked to complete the questionnaire immediately after each task.

To investigate usability, our research group collected spontaneous feedback and observation from participants' understanding of task instructions and interaction. In addition, the profile for technology use among participants was investigated using a Technology Use Profile Questionnaire (Box 1) developed by our research group. The questionnaire included 10 questions regarding routine use of technology devices and programs, such as smartphones, computers and video-games. This questionnaire was given to participants prior to task performance in order to investigate a possible influence of technology familiarity on the understanding and performance of both tasks and was not used for sample selection.

### The SOIVET system

The SOIVET system is a computer-based information system that utilizes an easy-to-use and low cost device for immersive virtual reality - the Samsung Gear VR™ headset, which is a mobile virtual reality headset developed by Samsung Electronics, in collaboration with Oculus, and manufactured by Samsung. It is compatible with the smartphone Galaxy S6/S6 Edge®, which acted as the headset's display and processor, while the Gear VR unit itself acted as the controller, which contains the high field of view, as well as a custom inertial measurement unit (IMU), for rotational tracking, and connects to the smartphone via micro-USB. For commands and movements used in the tasks, a Bluetooth joystick compatible with the Samsung smartphone was used. Both SOIVET-Maze and SOIVET-Route tasks, as well as the system's home menu were developed using the Unity® platform.

### Proposed tasks

Two immersive virtual reality tasks for the assessment of spatial orientation were proposed: the SOIVET-Maze, which focuses on the evaluation of allocentric to egocentric spatial ability; and the SOIVET-Route, which focuses on visuospatial memory and topographical landmark recognition. Participants were invited to use the system with the headset, which could be adjusted to properly fit the participant's head and focus eyesight accordingly. All participants performed the tasks in a quiet room, sitting in a mid-back office chair with adjustable seat height. Participants were able to interact with and to move around the virtual environment using a Bluetooth compatible joystick. An investigator was present at all times during the experiment.

### SOIVET-Maze

The SOIVET-Maze was inspired by and is a continuation of the work of Morganti et al., who developed a non-immersive virtual reality maze for the ecological assessment of spatial orientation abilities in the elderly population.^23^ The task is based on the traditional Money Road Map Test (MRMT) of sense of direction and was designed to investigate the transition from allocentric to egocentric spatial orientation - a crucial aspect of one's orientation in real life - which has shown to be specifically impaired in Alzheimer's Disease and not in healthy elderly.^23^^,^ 24 The SOIVET-Maze aimed to transfer the 3D interaction display for the Samsung Gear VR™ immersive interaction system and to apply automatically recorded and extractable data to the originally designed task.

**Figure 1:**
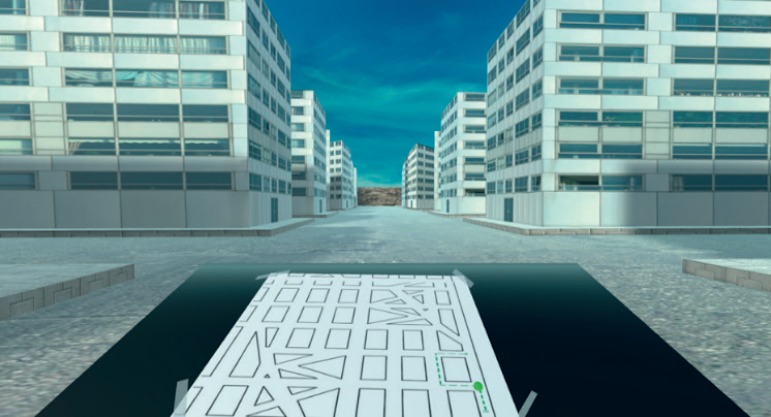
Participant's view in practice trial of SOIVET-Maze.

In the MRMT, participants are required to describe verbally each right or left turn from a depicted route on a paper map. A total of 32 turning points are presented in the MRMT' map, and all participants performed the original paper version prior to the VR experience. In SOIVET-Maze, participants are able to see the original route depicted on the map within the immersive virtual environment, but also to experience, from a first-person perspective, each turn in the maze (Figure 1). Participants are supposed to find their way in the VR maze, using the original MRMT map as the reference. The maze does not present any distinguishable landmarks, and participants can only use the map for orientation (Figure 1). A green point marks the last correct turn on the map, in order to reduce working memory effort.

Participants could not choose a wrong turn more than three times, and each mistake led back to the last turning point. All participants were able to perform a practice trial prior to task performance and to repeat the task once. The scoring system was based on the total of correct turns of participant's last trial performance. Akin to the MRMT scoring system, the maximum possible score is 32 points. There was no time limit.

### SOIVET-Route

**Figure 2:**
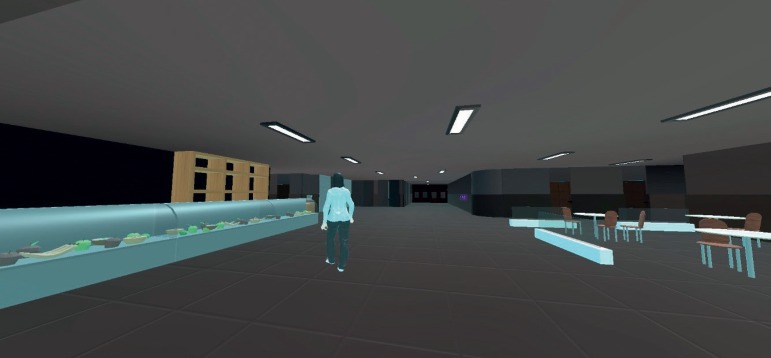
Participant's view of the avatar in Instructions phase of SOIVET-Route.

The SOIVET-Route task is based on the “Route” subtest from the Rivermead Behavioral Memory Test (RBMT).^25^ In the original version, the subtest requires participants to perform, and then recall, a route inside a health professional's office. The route includes five different landmarks: two chairs, a window, a table and a door. This particular subtest involves spatial orientation abilities, especially visuospatial memory, and has proven to be sensitive for detecting differences between Alzheimer's Disease patients, MCI patients, and healthy elderly.^26^ In SOIVET-Route, participants were instructed to follow an avatar and to perform, from a first-person perspective, a similar route, but in a larger area in the form of the entrance hall of a hospital building. After route performance following the avatar in the Instructions phase (Figure 2), participants were asked to perform the same route alone, immediately and after a 20-minute interval (delayed). All participants were allowed to repeat the instructions phase once if they wished. Akin to the original RBMT, the scoring system was based on the total of correct locations performed by the participants in the correct order. There was no time limit.

### Statistical analysis

To analyze User Experience outcomes data, a normal distribution analysis was performed, using skewness and kurtosis values as well as histogram visual checks. For additional evaluation, a Kolmogorov-Smirnov normality test was performed. Once the type of distribution was established, a paired sample t-test was used to compare means of parametric data and a Wilcoxon signed-ranked test to compare means of non-parametric data. To investigate correlation between data results, the Spearman correlation test (for non-normal distributed data) was used.

## RESULTS

### Sample characteristics

Participants were aged 18 to 59 years (M=32, ± 10.39 y), 54.8% women. All 31 participants had at least 12 years of education and 13 participants (41.9%) had 16 years or more. Mean years of education was 16.06 (±2.22), ranging from 12 to 20 years. None of the participants had auditory impairment and 61.3% had a visual refractive error (myopia or astigmatism), corrected with eyeglasses. Most participants (93.54% n=29) had a CCI of zero, two participants (6.45%) had a CCI of one, and one participant (3.22%) had a CCI of two. Technology use profile scores ranged from 18 to 39 (M=27.75, SD=5.967), where 40 was the maximum possible score. All 31 participants fulfilled inclusion criteria and no participants were excluded from the study prior to task performance.

### Tolerability

No significant tolerability differences were found between the SOIVET Maze task (mean value of 4.19±5.57) and the SOIVET Route task (3.52±6.42); Wilcoxon signed-rank test (Z= -.901, p= 0.368) - despite a small numeric difference in favor of the SOIVET-Route - as measured by the Cybersickness Questionnaire after each task performance. In addition, two (6.45%) participants were unable to complete the SOIVET-Maze task due to tolerability issues, while all participants were able to complete the SOIVET-Route task.

Cybersickness scores, from the Cybersickness Questionnaire (Box 3), on both tasks appeared to be significantly related to personal vulnerability and motion sickness history, since a positive correlation was found between the SOIVET-Maze and SOIVET-Route cybersickness scores (r=0.552, n=31, p=0.001) and between cybersickness scores on both tasks and our Motion Sickness Screening Questionnaire scores (r=0.533, p=0.002 and r=0.365, p=0.043 for SOIVET-Maze and SOIVET-Route, respectively). Cybersickness scores did not yield a significant correlation with gender (r= -0.341, p=0.061 and r= -0.116, p=0.533 for SOIVET-Maze and SOIVET-Route), age (r= 0.250, p=0.176 and r= -0.018, p=0.924 for SOIVET-Maze and SOIVET-Route) or technology use profile (r= -0.183, p=0.323 and r= -0.153, p=0.413 for SOIVET-Maze and SOIVET-Route).

### Sense of presence

Results indicated high levels of presence for both tasks, as measured by the Witmer and Singer Presence Questionnaire^22^ (Median scores of 128 and 143 for SOIVET-Maze and SOIVET-Route, respectively). Additionally, all 22 items from the questionnaire showed a median of 5 or higher on a 7-point Likert Scale for both tasks, where 7 indicates the highest sense of presence. Presence scores did not correlate with sex (r= -0.050, p=0.798 and r= -0.189, p=0.309 for SOIVET-Maze and SOIVET-Route, respectively), age (r= 0.007, p=0.971 and r= 0.175, p=0.348 for SOIVET-Maze and SOIVET-Route) or technology use profile (r= 0.016, p=0.936 and r= -0.036, p=0.846 for SOIVET-Maze and SOIVET-Route). Participants that were unable to complete the SOIVET-Maze due to tolerability issues (n=2, 6.45%) were excluded from the SOIVET-Maze presence score.

### Usability - understanding of task instructions and interaction

All participants were able to understand both task instructions and how to interact with the virtual environments using the controller joystick. The majority of participants required a second trial for performing the SOIVET-Maze task and a second trial for the SOIVET-Route instructions phase, due to an attention bias of novelty within the virtual environment. None of the participants required a second trial for the phases of immediate and delayed route recall in the SOIVET-Route. Additionally, technology use profile, as measured by the scores on our Technology use profile Questionnaire (Box 1) showed no correlation with performance on either task (r= -0.075, p=0.699; r= -0.286, p=0.118 and r= -0.265, p=0.150 for SOIVET-Maze and SOIVET-Route immediate and delayed recall, respectively).

## Discussion

The advantages of immersive virtual environments for the ecological assessment of cognitive processes hinge on the capacity of this technology to reproduce real-world sensory perceptions and to engage brain activation closely related to real life.^14^^,^ 16 It has been discussed that spatial orientation involves cognitive processes that are not engaged in traditional vista scale spatial assessments.^27^ Ecological evaluation - as proposed in VR tasks - may, therefore, reproduce neuronal mechanisms involved in everyday spatial orientation and closer resemble real-life. However, when seeking this kind of cognitive engagement, it is important to investigate whether patients experience a significant sense of presence in the virtual environment. This study was able to demonstrate that, besides being able to easily understand task instructions and interact with the virtual environments, both SOIVET-Maze and SOIVET-Route tasks seem to induce a strong sense of presence, which is important for ecological tasks.

Cybersickness, described as a type of motion or simulator sickness, is believed to result from a conflict between visual stimuli - which are highly enhanced within immersive virtual environments - and other sensory stimuli such as auditory, vestibular and proprioceptive information.^28^^-^ 30 The involvement of different sensory inputs, in particular, the vestibular system, has led to the use of VR environments for vestibular disorder rehabilitation.^31^ An important approach to prevent cybersickness in virtual environments is the ability to track the user's head position and to represent it accurately in the virtual space.^28^ Head-mounted displays, akin to that used in this study, have developed over the years, and are now able to provide this kind of information to the VR system. However, head-mounted displays can add other conflicting visual information that may worsen cybersickness, such as lags between actions performed and reproduced in the VR environment, position tracking errors, and conflicting depth perception.^28^

Apparently, different factors related to the individual, such as age, sex and ethnicity, can also contribute to one's susceptibility to motion sickness, as well as affective states such as anxiety.^30^^,^ 32 ^,^ 33 Interestingly, results of a strong correlation between tolerability scores from both tasks and a moderate correlation with motion sickness history found in this study points to the impact of an individual vulnerability factor.^34^ Since the SOIVET-Maze had a higher correlation score with motion sickness history, it is likely that this task in particular was causing motion sickness related effects. An individual vulnerability factor supports the need to carry out an active assessment of motion sickness or cybersickness history of all participants prior to VR tasks performance. Researchers - and perhaps in the future, clinicians - should try to minimize the emergence of severe adverse events by screening susceptible patients. It is important to assess, and as far as possible minimize, tolerability issues in immersive virtual environments, especially when applied to health care. Our results indicate that both tasks had no statistical difference in tolerability profile, but a numeric advantage for the SOIVET-Route was noted. A better understanding of technical features presented in the SOIVET-Maze compared to the SOIVET-Route could help elucidate this tolerability difference and allow some improvements: a major difference between the two tasks is the way participants move around the virtual environment. In the SOIVET-Maze, participants had the perception of moving around the maze inside a vehicle. To turn right or left, they could do so only by using the controller joystick. In contrast, to move around the hospital entrance hall in the SOIVET-Route, participants had the perception of walking and were only able to turn right or left by rotating their heads in the chosen direction, in addition to the controller. Thus, movement in the SOIVET-Maze was faster and independent of head turns (i.e. independent of head position tracking). Other contributing factors could have been the uncomfortable landscape perception (a tight maze vs a spacious hospital entrance hall), and possible conflicts between the perception of landscape proportion from vista scale (map) *vs* large scale.

At the initial phase of an immersive task development, it is important to assess User Experience measures for a better understanding of negative features of tasks and ways to refine them.^35^ We believe the present study can provide some examples of tolerability, sense of presence and usability measures, which may be helpful for other research groups focused on health-applied technology assessment. One limitation of this study was the recruitment of younger adults, instead of elderly. Although assessing User Experience with the target group would be ideal, in the case of elderly, we believe it was preferable to obtain these measures in a younger group, in order to adjust and improve task usability and comfort. However, it is important to investigate User Experience measures among elderly, once the tasks are considered ready to use. Results for tolerability profile, as well as understanding of task instructions and operation, could differ in elderly relative to results obtained for adults. Since Sense of Presence did not correlate with age, gender or technology use profile, this aspect is not expected to vary significantly among older participants, but should also be investigated.

In conclusion, the SOIVET-Maze and SOIVET-Route seem to be well-tolerated, engaging and easy-to-use immersive tasks. The present study yielded favorable results that encourage further investigation of the proposed tasks in different population and patient profiles.
